# Changes of stress oxidative enzymes in rat mammary tissue, blood and milk after experimental mastitis induced by E. coli lipopolysaccharide

**Published:** 2015-06-15

**Authors:** Hadi Eslami, Rooz Ali Batavani, Siamak Asr‎i-Rezaei, Rahim Hobbenaghi

**Affiliations:** 1 *Department of Theriogenology, Faculty of Veterinary Medicine, Urmia University, Urmia, Iran; *; 2 * Department of Internal Medicine and ClinicalPathology, Faculty of Veterinary Medicine, Urmia University, Urmia, Iran; *; 3 *Department of Pathobiology, Faculty of Veterinary Medicine, Urmia University, Urmia, Iran.*

**Keywords:** Antioxidant enzymes, Lipopolysaccharide, Malondialdehyde, Mastitis, Rat

## Abstract

The present study investigated the effects of *E. coli* lipopolysaccharide (LPS) induced mastitis model in rat on the activity of antioxidant enzyme systems. To achieve this purpose, *E. coli* LPS were infused into the mammary gland of 12 rats 72 hr postpartum and compared with 12 rats in control group infused intramammary placebo sterile pyrogene – free, physiological saline. The antioxidant activities of the enzymes, superoxide dismutase, glutathione peroxidase, and catalase together with ‎‏malondialdehyde (MDA) level were assayed in blood serum, milk and mammary tissue. Results obtained showed that, the antioxidant enzyme activities in milk, blood serum and mammary tissue were significantly decreased while the level of MDA, the indicator of lipid peroxidation were significantly increased following intramammary inoculation of LPS compared to the control animals. Histopathological examination also revealed the infiltration of inflammatory cells in mammary tissue and disruption of acinar structure and acinar lumina in mastitic rats. The results indicated that *E. coli* LPS-induced ‎mastitis could alter antioxidant enzymes and increase lipid peroxidation.

## Introduction

Mastitis can affect all lactating mammals, and it is the most important disease of the mammary gland in dairy cows, with ‎‎economic implications, due to losses in milk production and the risk posed by consumption of ‎‎infected milk to public health.

A few hours after the infection of the udder with pathogenic ‎microorganisms, the number of somatic cells (SCC) in milk increase in ‎response to ‎activation of inflammatory processes. Macrophages play an important role ‎in overseeing ‎infected gland. When the bacteria invade and colonize the mammary gland, macrophages ‎respond by initiating the inflammatory response, one that attracts ‎polymorphonuclear cells ‎in milk to kill the bacteria. More than 90.00% of SCC in ‎infected glands are composed of ‎neutrophils.^1^ The antibacterial activity of neutrophils is ‎mediated via reactive oxygen species (ROS). Various infectious diseases of farm animals, such as pneumonia, enteritis and ‎mastitis are associated with oxidative ‎stress. Although ‎oxidative reactions are essential for the body, an excess of ‎oxidative reactions of ‎the anti-bacterial processes may cause tissue damage. An excess of ROS ‎and the absence of ‎optimal amounts of antioxidants are leading to oxidative stress. Many cells ‎are susceptible to ‎this oxidative stress, which can cause necrosis or apoptosis.^2^^,^^3^ Different enzymes can ‎prevent the formation of radicals or scavenge radicals or ‎hydrogen peroxide and other ‎peroxides. Among antioxidant enzymes, superoxide dismutase and ‎catalase have been ‎demonstrated in milk.^4^ Superoxide dismutase (SOD) is considered the most important enzyme and the presence of this enzyme is important in maintaining the antioxidant stability ‎of milk. ‎Studies have shown that exogenous addition of SOD causes a reduction in lipid ‎peroxidation ‎processes, providing greater stability of milk‎.^5^ Catalase (CAT) is an enzyme that ‎catalyzes ‎the decomposition of hydrogen peroxide. In milk, the enzyme can be both mammary ‎‎gland and bacterial origin, is heat labile and are inactivated within minutes at 65 ˚C. ‎Its ‎absence in milk shows that it has been properly pasteurized. Present in large quantities, ‎‎catalase is an indication that the udder has held an inflammatory process. It was demonstrated that catalytic activity can be used as ‎a marker of ‎mastitis.^6^

Another enzyme in milk with antioxidant functions is the ‎glutathione ‎peroxidase. The enzyme glutathione peroxidase (GPx) is a family of enzymes play important ‎roles in the ‎protection of ‎organisms from oxidative damage that converts reduced ‎glutathione (GSH) to ‎oxidized ‎glutathione (GSSG) while reducing lipid hydro-peroxides to their corresponding ‎alcohols or ‎free hydrogen peroxide to water.^7^^,^^8^

Coliform bacteria (including Escherichia coli, Klebsiella species, and Enterobacter species) ‎are the most common causative agents in severe clinical cases of mastitis.

Approximately 80.00% ‎of all intra mammary infections by coliform bacteria result in clinical mastitis.^9^^,^^10^ All of ‎these gram-negative bacteria contain lipopolysaccharide (LPS) in their outer cell membranes. ‎The mammary gland is highly sensitive to LPS,^11^^,^^12^ and LPS-induced models of mastitis ‎are valuable tools to study the effects of coliform mastitis because they mimic the responses ‎observed during natural mastitis without the risks associated with a bacterial infection.^13^^,^^14^

Since the bovine model is expensive, the use of an appropriate, sensitive, and ‎reliable animal model is essential for the experimental study of mastitis. A mouse mastitis ‎model, initially introduced by Chandler,^15^ has been used for studying coliform ‎mastitis.^16^ However, for mouse mammary gland inoculation, the tip of the teat must be ‎amputated.^17^ In contrast, the teat canal and the mammary gland of lactating rats are larger ‎than those of mice and inoculation can be reliably reproduced. The endotoxins are the key ‎molecules in the induction of inﬂammation and the inflammatory response in the animal. ‎Furthermore, pathophysiologically, the response is dose-dependent.^18^^,^^19^ It is therefore ‎possible to induce experimental coliform mastitis by intramammary (IM) infusions of ‎LPS instead of intact, living bacterial cells – a “sterile and non-infectious” technique. The ‎technique has been used extensively for decades because the administration of LPS induces ‎the same local signs as observed during E. coli mastitis‏.‏ To the best of our knowledge, the correlation between antioxidant enzymes activity in milk, serum ‎and mammary tissue has not yet been fully considered. Therefore, the aim of the present ‎study was to examine possible relationships between milk, serum and mammary tissue ‎oxidative stress enzymes and oxidative stress in rat model experimental induced mastitis.‎

## Materials and Methods

‎Materials. Escherichia coli lipopolysaccharide (LPS, ‎O55:B5, Sigma Chemical Co., St. Louis, USA) was diluted in sterile pyrogen-free physiological saline and adjusted to a ‎concentration of 0.1 mg mL^-1^.‎

Animals and treatments. The experimental and animal care procedures were approved by the Veterinary ‎Ethics Committee of the Faculty of Veterinary Medicine of Urmia University and were ‎performed in accordance with the national institutes of health guide for care and use of ‎laboratory animals. Twenty four healthy adult female rats of Wistar strains, weighting 200 to 250 ‎g, and 12 male rats, weighing 250 to 350 g, were used in this study. Male and female rats were ‎kept in a room with a controlled ambient temperature (22 ± 1 ˚C ‎‎), relative humidity 60 ± 10%, ‎under a 12 hr light-dark cycle (light on at 07: 00 AM) and gravitational mechanical ‎ventilation, separately. Commercial rat pellet diet and tap water were given ad libitum. They ‎were allowed to adapt to this condition for one week before mating. Two female rats and one ‎male rat were housed per cage with food and water ad libitum. Female rats were randomly ‎divided into two groups of twelve animals each after confirmation of mating (vaginal plug) ‎and pregnancy. Seventy two hours after postpartum milking rats were anesthetized with ether ‎and only the teat end of two rear quarters was sanitized with cotton swabs ‎soaked in 70% ethyl alcohol. The rats of the positive control were infused slowly with 10 μg ‎of E.coli LPS dissolved in 100 μL of sterile and pyrogen-free, ‎physiological saline into the inguinal mammary glands (left and right fourth glands). This dose ‎of LPS ‎(0.1 mg per 1 mL)‎ has been shown to reliably cause mastitis.^20^ Twelve rats in control group were ‎infused with an equal volume (100 μL) of normal saline. The LPS and saline solutions were infused into the glands ‎with a sterile 33-gauge needle fitted to a 1 mL syringe and quarters were thoroughly ‎massaged. Intramammary infusion was according to the method of Zhong et al.^21^ The pups were ‎weaned 72 hr prior to experimental inoculation and reared with surrogate milking rats. Rats ‎were observed till 12 hr post LPS and saline infusions and mastitis were assessed in terms of ‎clinical appearance, macroscopic and microscopic changes of the mammary glands. For the 12 rats, positive diagnosis was confirmed by swelling, redness and pathological ‎changes (poly-morphonuclear cells infiltration, acinar structure and acinar lumina disruption)‎.‎

Milk collection. To stimulate milk let down, oxytocin was prepared as a 4 IU mL^-1 ^solution and ‎administered ‎intra-peritoneally at a dose level of 4 IU kg^-1 ^body weight, at a standard dose volume ‎of 1 mL kg^-1 ^‎body weight.^22^^,^^23^ Teat ends were swabbed with cotton wool soaked in 70% ‎ethyl ‎alcohol and milk samples were collected using a vacuum milking system.^24^ Milk samples were ‎collected 12 hr after LPS infusion in sterile test microtubes (1 mL), and samples were defatted by centrifugation ‎‎(Model 1-14 microfuge; Sigma, Osterode, Germany) at 2,500 g for 15 min and the ‎skimmed milk was used for further analysis. ‎Samples stored at – 20 ˚C until enzyme analysis.‎ Enzymatic assays were perfomed using the commercial kit (Cayman Chemical Co., Ann Arbor, USA) ‎according to ‎the ‎manufacturer’s protocol. ‎

Preparation of blood serum. After milking, animals were anesthetized deeply by ether inhalation and then euthanized ‎by ‎draining blood from their hearts, followed by cervical dislocation. Blood samples ‎were ‎centrifuged at 2,000 g for 10 min at 4 ˚C ‎‏)‏Model 5804 centrifuge; Eppendorf AG, Hamburg, Germany‎)‎ to obtain serum and stored in – 20 ˚C until enzyme analysis.‎ The procedures were ‎performed according to ‎the ‎manufacturer’s protocol like ‎the mammary tissues supernatant. ‎

Preparation of mammary tissue. After milking, animals were anesthetized deeply by ether and subsequently euthanized and mammary tissue dissected in aseptic condition. Mammary tissues were washed with small amount of saline and divided in four parts and every part was weighed. The enzyme level in rat mammary tissues was measured using the assay kits ‎‎(Cayman Chemical Co., Ann Arbor, USA). The remaining ‎mammary tissue was fixed in 10% neutral-buffered formalin and used for histological observation.

Histological observation. Tissue specimens were fixed in 10% neutral buffered formalin for 24 hr. Dehydrated tissues ‎were then embedded in paraffin. Sections were stained with hematoxylin and eosin. ‎Histological changes were observed by light microscopic examination (Model BH2; Olympus Optical, Tokyo, ‎Japan) at 200 × magnification.

Catalase assay. The catalase level in rat mammary tissues was measured using the CAT assay kit (Cayman Chemical Co., Ann Arbor, USA). The experimental procedures were carried out according to the ‎manufacturer’s ‎protocol. Briefly, the mammary tissues were homogenized with polytron homogenizer (Model PT3100, Kinematica, Littau, Switzerland) in cold buffer (50 mM ‎potassium ‎phosphate, 1 mM EDTA, pH 7 per g of tissue). The supernatant was collected after ‎centrifugation at ‎12,000 g for 20 ‎min at 4 ˚C (Model 5804 centrifuge; Eppendorf, Hamburg, Germany)‎. The sample was mixed with diluted assay buffer and ‎methanol. The reaction was initiated by adding diluted hydrogen ‎peroxide for 20 min with constant ‎shaking. Diluted potassium hydroxide was then added followed ‎by catalase purpald. The plate was ‎incubated immediately for 10 min with constant shaking. ‎Catalase potassium periodate was then ‎added followed by 5 min incubation with constant ‎shaking. The absorbance was then read at 540 ‎nm with a spectrophotometer (Cary 300, Varian Co., Palo Alto, USA ‎).‎

Superoxide dismutase assay. The SOD level in rat mammary tissues was measured by SOD assay kit (Cayman Chemical Co., Ann Arbor, USA) according to manufacturer’s ‎protocol. Briefly, the mammary tissues were homogenized with the polytron homogenizer in 20 mM N-2 hydroxy-ethyl piperazine-N'-2-ethanesulfonic acid (HEPES buffer), 1 ‎mM ethylene glycol tetra-acetic acid, 210 mM mannitol, 70 mM sucrose, pH 7.2  per g of tissue). The supernatant was ‎collected after ‎centrifugation at ‎12,000 g ‎(Model 5804 centrifuge; Eppendorf, Hamburg, Germany) for 20 min at 4 ˚C‎. The reaction was initiated by adding diluted xanthine ‎oxidase. The plate was incubated immediately for 20 min with constant shaking. The absorbance ‎was then read at 450 nm ‎with the spectrophotometer.‎ ‎

Glutathione peroxidase (GPx)‎ assay. The glutathione peroxidase level in rat mammary tissues was measured using the GPx assay ‎kit ‎ (Cayman Chemical Co., Ann Arbor, USA). The experimental procedures were carried out according to ‎the ‎manufacturer’s protocol. Briefly, the mammary tissues were homogenized with the polytron homogenizer in cold buffer ‎(50 mM ‎Tris-‎HCL, PH 7.5, 5 mM ‎EDTA, and 1 mM dithiothreitol)‎ per g tissue and then centrifuge at 10,000 g for ‎‎15 ‎min at 4 ˚C ‎. The supernatant was collected after centrifugation.‎ Nicotinamide adenine dinucleotide phosphate (NADPH) and glutathione ‎reductase (GR) were reduced to glutathione (GSH). Therefore, the rate ‎of ‎NADPH consumption was ‎utilized as a measurement of the rate of glutathione disulfide (GSSG) formation‎. The ‎supernatants from tissue ‎homogenates were mixed with the stock solution containing‎ ‎NADPH, GSH, and excess GR and ‎incubated at 37 ˚C for 5 min, followed by addition of 20 μL of ‎cumene hydroperoxide as a substrate.‎ ‎The absorbance was then read at 340 nm with an absorbance ‎reader ‎(Varian Co., Palo Alto, USA‎).‎ ‎

Malondialdehyde (MDA) assay.‎ The level of MDA in rat mammary tissues was measured using thiobarbituric acid reactive substances (TBARS) assay ‎kit (Cayman Chemical Co., Ann Arbor, USA). The experimental procedures were carried ‎out ‎according to the ‎manufacturer’s protocol. Briefly, tissue was ‎sonicated ‎in radioimmunoprecipitation assay (RIPA) buffer for 15 ‎sec at 40 V over ice‎, centrifuged at ‎‎1,600 g for 10 min at 4 ˚C. Assay was ‎performed ‎according to manufacturer's directions. The absorbance was then read at 532 nm with ‎the a spectrophotometer.

Statistical analysis. Data were analysed by SPSS (Version 18; SPSS Inc., Chicago, USA). Studentʼs ‎ t-test was carried out to find the differences between the results of mastitic and ‎non-mastitic milk, serum and mammary tissue homogenate. The results are expressed as mean ± ‎SEM.

## Results

Following tests carried out on the 24 lactating rats, 12 of them had positive ‎diagnosis. ‎Results obtained from analysis of antioxidant enzyme activity in milk, blood samples ‎and mammary tissue (mean ± ‎SD) are presented in Table 1. The level of SOD‎‎, CAT and GPx in milk, serum and tissue in experimental mastitic rat compared to ‎control ‎rat showed significant decrease (p‎ <‎ 0.05) ‎and the level of MDA showed marked elevation (p ‎< 0‎.05) in comparison ‎with control rats.

**Table 1:** Oxidative stress parameters in rats infused by LPS (n = 12) and in healthy rats (n=12) ‎in serum, milk and mammary tissue supernatant. Results are expressed as mean ± standard ‎deviation.

**Parameters** *	**Control group**	**Mastitic group**	***p*** **-value**
**SOD:** **Mammary tissue (U mg**^-1^ **‎)**	21.70 ± 0.27	21.13 ± 0.21	*p*‎ < 0‎.05
**SOD: Milk (U mL** ^-1^ **)**	4.14 ± 0.12	3.84 ± 0.11	*p*‎ < 0‎.01
**SOD: Serum (U mL** ^-1^ **)**	2.06 ± 0.05	1.87 ± 0.09	*p*‎ < 0‎.01
**GPx: Mammary tissue (nmol mg** ^-1^ ** per min)**	29.64 ± 0.51	23.46 ± 0.20	*p*‎ < 0‎.01
**GPx: Milk (nmol mL** ^-1^ ** per min)**	19.38 ± 0.23	15.40 ± 0.24	*p *‎< 0‎.01
**GPx: Serum (nmol** **mL**^-1^** per min)**	29.81 ± 0.34	26.35 ± 0.22	*p *‎< 0‎.01
**CAT: Mammary tissue (μmol mg** ^-1^ **‎)**	22.00 ± 0.35	18.41 ± 0.17	*p*‎ < 0‎.01
**CAT: Milk ** **‎** ** (** **μmol mL** ^-1^ **‎)**	1.52 ± 0.00	1.42 ± 0.00	*p*‎ < 0‎.01
**CAT: Serum ** **‎** ** (** **μmol mL** ^-1^ **)**	1.78 ± 0.01	1.50 ± 0.00	*p*‎ < 0‎.01
**MDA: Mammary tissue (** **‎** **μM mg** ^-1^ **)**	26.45 ± 0.20	47.15± 0.40	*p*‎ < 0‎.01
**MDA: Milk (μM mL** ^-1^ **)**	30.83 ± 0.38	41.87 ± 0.39	*p *‎< 0‎.01
**MDA: Serum (μM mL** ^-1^ **)**	14.13 ± 0.17	28.02 ± 0.30	*p *‎< 0‎.01

* SOD = Superoxide dismutase, GPx = Glutathione peroxidase, CAT = Catalase, and ‎‏ MDA = Malondialdehyde.

**Fig. 1:**
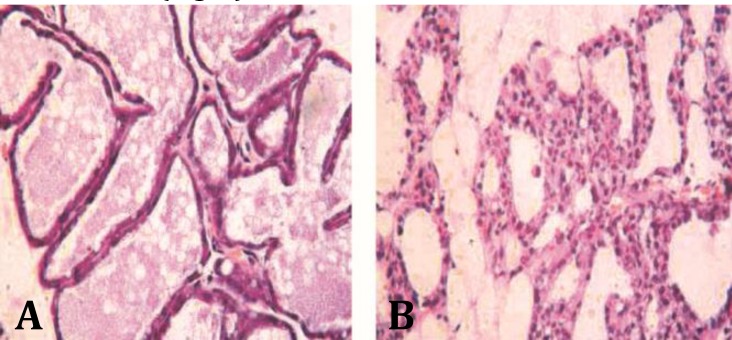
Histopathology of rat mammary gland at 12 hr after intra-mammary infusion LPS, (A) control group: Mammary gland tissue, epithelial tight junction, intact acinar structure and no infiltration of inflammatory cells in mammary gland, (B) LPS infused group: inflammatory cells were infiltrated in mammary tissue, acinar structure and acinar lumina were also disrupted

Histopathological examination revealed the infiltration of inflammatory cells in mammary tissue and disruption of acinar structure and acinar lumina in mastitic rats (Fig. 1).

## Discussion

Although laboratory changes are well examined in many diseases in rats, ‎oxidative ‎stress ‎parameters induced by LPS are poorly studied. Also, ‎oxidative statuses of rat ‎mastitis ‎induced by LPS are not ‎investigated. Many potentially toxic ROS ‎are generated ‎through ‎normal oxidative metabolism and the body has adapted ‎by ‎developing ‎a ‎complex system of protective antioxidants.^25^ Oxidative ‎stress may be ‎defined ‎as an ‎alteration in the steady-state balance between ‎oxidant and ‎antioxidant agents in the ‎cells. ‎With increase of intracellular sources of ‎ROS, several physiological processes may ‎be ‎disturbed.^26^ Under ‎more stressful conditions such as mastitis, hydroxy radicals‎ released ‎by ‎‎infiltrated neutrophils caused mammary cell injury. This ‎peroxidation ‎associated mechanism ‎was demonstrated in an invitro model ‎where antioxidants were proved useful to prevent ‎mammary ‎tissue damage ‎during ‎bovine mastitis.^27^ ‎Antioxidant enzymes and the ‎determination ‎of ‎ MDA ‎concentrations are among the most widely used ‎‎methods for ‎determination of ‎oxidative stress.^28^

Increased plasma MDA ‎‎concentration is considered as ‎a ‎marker of lipid peroxidation.^29^ In the present study, ‎values of GPx, SOD and CAT significantly were decreased in milk, serum ‎and tissue ‎in mastitic rat, however, MDA levels were increased significantly. ‎In similar studies El- Deeb, reported the same in ‎milk of mastitic does and Li-Li and Qingzhang, in milk and mammary ‎tissue of goat with gangrenous mastitis showed ‎that the level of GPx, SOD and CAT ‎were ‎decreased significantly and the level of MDA was significantly ‎increased when compared to ‎control animals.^30^^,^^31^ On the other hand, Atroshi et al. in cows with mastitis and ‎kizil et al. in goat with ‎M. agalalactia infected mastitis showed high level of MDA and reduction of GPx ‎activity in ‎blood.^32^^,^^33^ Also, Yang et al,^34^ showed high level MDA and SCC and decreased in GPx activity in ‎milk ‎that is similar with our results.

In this study, MDA concentrations were found to be increased in the ‎serum, milk and tissue ‎of rats ‎with mastitis compared to the control group, ‎while decrease of GPx, SOD and CAT ‎activities were ‎observed.‎ These changes ‎were coupled to the increase of MDA concentrations ‎confirmed the occurrence of ‎an ‎oxidative stress during mastitis caused by LPS. Increased lipid peroxidation as a result of ‎changed‏ ‏intracellular ratio between the free radicals ‎and antioxidant system has been suggested ‎to be correlated‏ ‏with mastitis. These ‎findings are in agreement with those ‎obtained by Atroshi et al. in cows and Çetin et al. in ewes.^35^

The higher ‎MDA levels in mastitic rats reported ‎ in this ‎study demonstrated ‎that ‎the auto-oxidative ‎activity of mastitic case is ‎higher than normal.‎ Because during ‎inflammation‎, oxidation of ‎long chain fatty acids in cell membranes lead to ‎lipid peroxidation,^36^^,^^37^ which may inhibit the ‎activity of ‎some antioxidant mole‏cules as ‎‎GPx leading to oxidative ‎stress.^38^ The ‎present‏ ‏results indicated that increasing levels of oxidative‏ ‏stress markers in rats ‎mastitis ‎might‏ ‏have an essential role in the process of inflammation‏ ‏and tissue damage and‏ ‏MDA ‎is the final product of lipid ‎peroxidation and ‎therefore is used as index of ‎‎ this ‎process. ‎

These changes in enzyme ‎activities in blood or other‏ biological fluids such as ‎milk ‎ could be consequence of‏ ‏‎ cell structural damage and may indicate ‎that GPx, SOD ‎and CAT levels were ‎depressed when lipid peroxidation was increased and Low GSH-Px activity in ‎mastitic cows with high ‎SCCs and high level of prostaglandin formation has ‎been recorded,^39^ which may be ‎attributed to the excessive release of free ‎radicals that may ‎result in inhibiting enzymes activity ‎and ‎lead to an exacerbation of the oxidative stress‎. On ‎the other hand, ‎decrease in enzymatic ‎antioxidant activities‏ ‏‎ might be attributed either ‎to the ‎increase in consumption or to‏ ‏the ‎counteraction with ROS produced ‎from‏ ‏inflamed ‎gland, suggesting a compromise in‏ ‏antioxidant ‎defence of ‎the body.‎‏
